# Objective assessment of flap volume changes and aesthetic results after adjuvant radiation therapy in patients undergoing immediate autologous breast reconstruction

**DOI:** 10.1371/journal.pone.0197615

**Published:** 2018-05-21

**Authors:** Yujin Myung, Yousung Son, Tae-hyun Nam, Eunyoung Kang, Eun-Kyu Kim, In Ah Kim, Keun-Yong Eom, Chan Yeong Heo, Jae Hoon Jeong

**Affiliations:** 1 Department of Plastic and Reconstructive Surgery, Seoul National University Bundang Hospital, Seongnam, Republic of Korea; 2 Department of Diagnostic Radiology, Seoul National University Bundang Hospital, Seongnam, Republic of Korea; 3 Department of Surgery, Seoul National University Bundang Hospital, Seongnam, Republic of Korea; 4 Department of Radiation Oncology, Seoul National University Bundang Hospital, Seongnam, Republic of Korea; Universita degli Studi di Roma La Sapienza Facolta di Medicina e Psicologia, ITALY

## Abstract

**Background:**

The use of immediate breast reconstruction and adjuvant radiation therapy is increasing in breast cancer patients. This study aimed to analyze the aesthetic outcome and changes in flap volume in patients with breast cancer undergoing radiation therapy of the surgical site after immediate autologous tissue reconstruction.

**Methods:**

Immediate abdominal free flap breast reconstruction following unilateral mastectomy was performed in 42 patients; 21 patients received adjuvant radiation (study group) and 21 patients did not (control group). To compare flap volume, three-dimensional computed tomography (CT) was performed before and after radiation. Also, aesthetic analysis was performed in both groups to evaluate shape changes.

**Results:**

There was a 12.3% flap volume reduction after the completion of radiation in the experimental group that was significantly greater than the 2.6% volume reduction observed in the non-radiation group (*P*<0.01). There was no significant difference in the short- and long-term aesthetic results between the groups.

**Conclusions:**

When performing immediate autologous breast reconstruction, 14% volume overcorrection is recommended for patients in whom adjuvant radiation therapy is anticipated to improve aesthetic outcomes.

## Introduction

Advances in the surgical and oncological management of breast cancer have optimized the use of breast cancer surgery with regard to patient well-being and postoperative aesthetics of the breast, as well as in the prevention of tumor recurrence and improved long-term survival [1]. Because of its many patient benefits, immediate reconstruction is being performed more frequently, and flap reconstruction methods using autologous tissue are being used in many patients [2].

When performing immediate breast reconstruction, it is difficult for the surgeon to anticipate changes in breast volume and shape over time, and most surgeons concentrate on immediate aesthetic results and symmetry. However, patients receiving adjuvant radiation therapy may experience worsening long-term aesthetic outcomes after breast reconstruction because of radiation-induced tissue damage and necrosis [3]. In addition, radiotherapy is often indicated after post-mastectomy reconstruction if surgical margins are inadequate or if microscopic tumor remains after the resection [4].

A few earlier studies have reported a higher rate of complications, such as flap shrinkage after radiotherapy, whereas more recent studies have found acceptable results [3, 5–10]. The authors believe that the main factors resulting in this discrepancy are advances in radiation therapy regimens and modalities, which enabled the delivery of more concentrated doses and allowed the preservation of nearby structures. Various efforts have been made to reduce complications by modifying treatment regimens, modalities, and positions [11,12]. However, most studies to date have focused on complications after radiotherapy [3, 5–12], primarily the occurrence of subjective symptoms. These studies have provided a limited assessment of the use of postoperative radiotherapy after flap or implant-based reconstruction and did not provide reconstructive surgeons with current information regarding the outcomes after radiotherapy. These studies have not assisted the surgeon in planning and implementing immediate reconstruction.

The purpose of this study was to determine the degree of flap volume decrease and to assess the morphological changes following irradiation of the reconstructed breast, to assist the surgeon in predicting the long-term outcome of immediate breast reconstruction. In this study, we compared the flap volume changes between a group of patients undergoing radiotherapy with those of a propensity-matched control group not receiving radiotherapy to analyze the clinical significance of volume reduction after radiotherapy in patients undergoing unilateral mastectomy and immediate autologous tissue breast reconstruction. We performed a quantitative and objective analysis of the effect of radiotherapy on flap volume using three-dimensional (3D) computed tomography (CT) imaging before and after surgery. In addition, using an objective scoring system, we compared the aesthetic results in patients undergoing radiation therapy after breast reconstruction with those of patients that did not receive radiation therapy.

## Patients and methods

### Patients

A total of 21 patients who underwent immediate breast reconstruction using a free transverse rectus abdominis (TRAM) or free deep inferior epigastric perforator (DIEP) flap at Seoul National University Bundang Hospital between 2012 and 2016 were enrolled in a retrospective cohort study. The treatment protocols of the patients were carried out per routine clinical care and there were no additional intervention to construct the present study. Adjuvant radiotherapy was done to the patients according to the staging of primary tumor, as the indication of radiation were patients with tumor size over 4 centimeters or if 4 or more positive axillary nodes were present. All patients underwent autologous reconstruction after unilateral mastectomy and all received adjuvant radiotherapy within 3–9 months after surgery. The radiotherapy protocol was 50 Gy in 25 fractions on the whole breast using the parallel opposed field technique. If necessary, 10–16 Gy in 5–8 fractions using 9–16 MeV electron was boosted on the tumor bed area. We used two linear accelerators (Clinac 21EX and Clinac 21EX (Silhouette), Varian, California, USA). In addition, a cohort of 21 patients who underwent immediate breast reconstruction through an abdominal free flap, who did not receive adjuvant radiation therapy, and who were propensity matched with regard to age, weight, type of mastectomy, resected weight, and weight of inserted flap were enrolled in the study as controls. In all surgeries, flaps contained more than two perforators, and no venous superdrainage was performed. We excluded patients with co-morbidities such as diabetes. We received Institutional Review Board (IRB) approval from the Seoul National University Bundang Hospital. Written Informed consent was obtained from all individual participants included in the study.

### Volumetric analysis

CT scan images obtained after the completion of radiation therapy were compared with images obtained between 12–18 months postoperatively in the radiotherapy group. In the control group, CT images were obtained between 3–6 months postoperatively and were compared with images obtained at 12–18 months postoperatively.

CT scans were performed using 64 multidetector computed tomography (MDCT) (Brilliance 64, Philips Healthcare, Cleveland, OH, USA) and 256 MDCT (Brilliance iCT, Philips Healthcare, Cleveland, Ohio, USA) machines. A 3D workstation (Aquarius iNtuition edition V 4.4, TeraRecon, Inc, San Mateo, CA, USA) was used to measure the total volume using the Digital Imaging and Communications in Medicine (DICOM) file for image analysis. Image data used in this study were obtained from a dedicated chest CT protocol for breast cancer patients that included the entire breast and total area of the reconstructed flap. Both 64 MDCT and 256 MDCT machines were set to a slice thickness of 2 mm and a slice interval of 1 mm.

**Fig 1:**
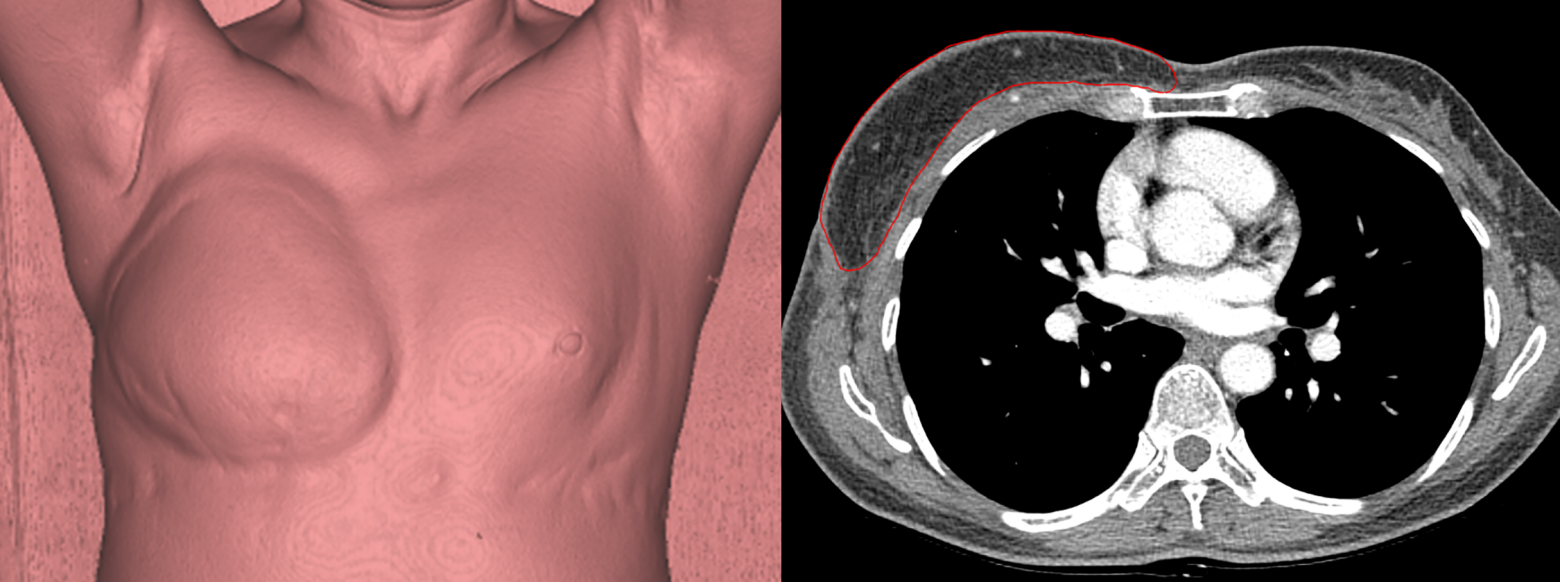
Volumetric analysis. (A) Three-dimensional reconstructed image of chest computed tomographic study. (B) Outline of abdominal flap was manually marked in each scan image.

**Fig 2:**
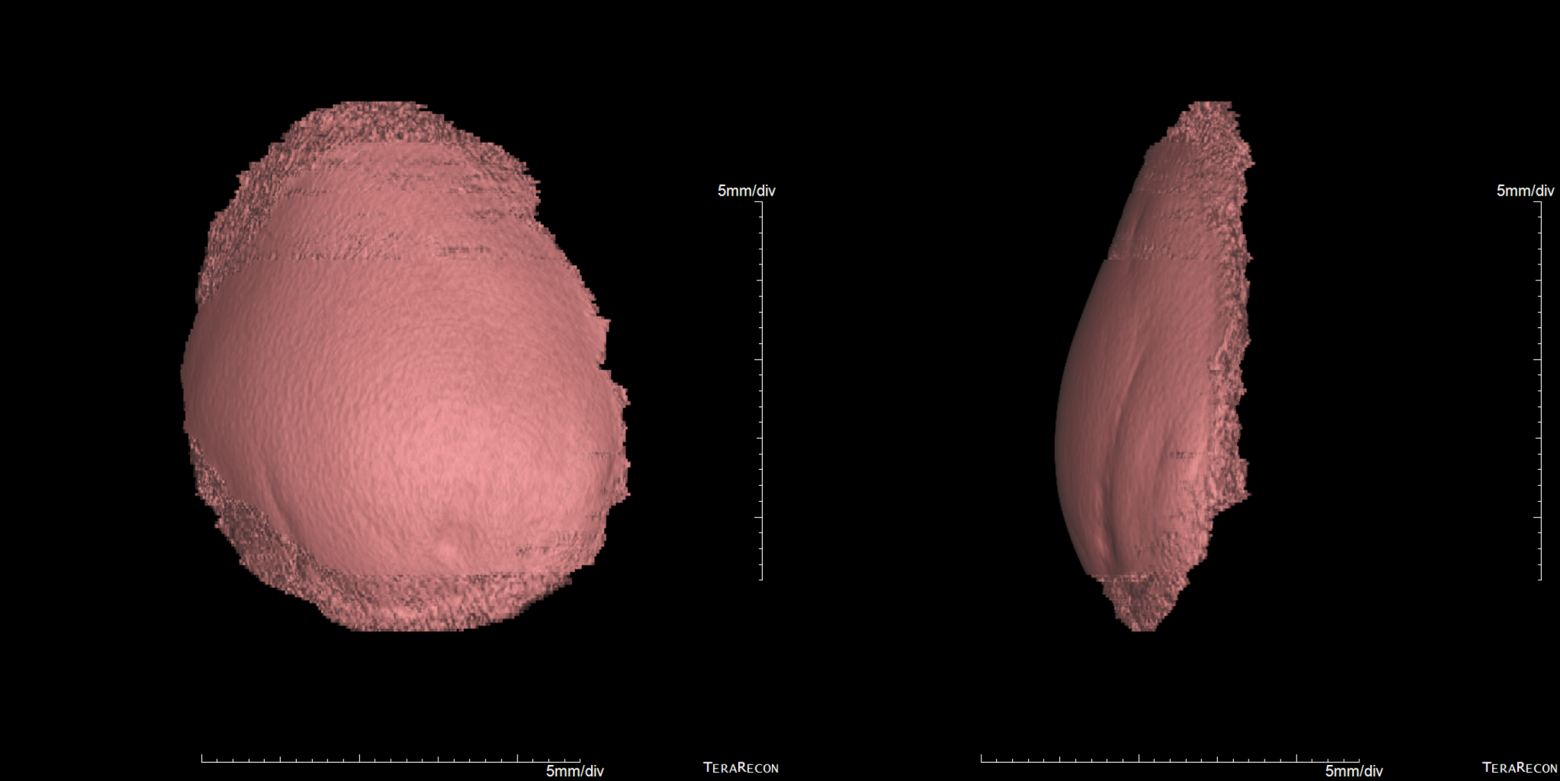
Reconstructed images of the flap. Anterior view and lateral view of reconstructed images.

To evaluate the volume of the flap, two independent physicians analyzed the CT images of the 42 patients. For the analysis of the DICOM images acquired by CT, the 3D-workstation's multi-planar reformation function was used to reconstruct the slice thickness of the breast region to 5 mm and the interval to 5 mm, and the measurements were performed on a CT image with a window center of 30 Hounsfield units (HU) and a window width of 400 HU in an axial image so that the target region could be visually confirmed. The data was loaded into the VolBrowse® and segmentation was performed by contouring the reconstructed site in a linear fashion using the free region of interest function on each slice (Fig 1). The total volume values of the segmented volume rendering image before and after radiotherapy were calculated and compared (Fig 2).

### Breast cosmesis analysis

**Fig 3:**
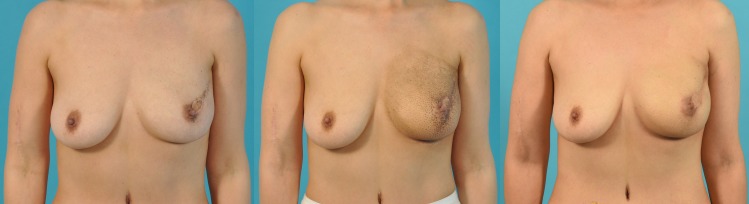
Photographic image of a patient. (A) 43-year old patient 4 weeks after nipple-sparing mastectomy and immediate breast reconstruction with a free transverse rectus abdominis flap. (B) After completion of adjuvant radiation therapy. (C) One year after the completion of radiation therapy. Increased symmetry and a decrease in the upper part of the reconstructed breast is observed.

**Fig 4:**
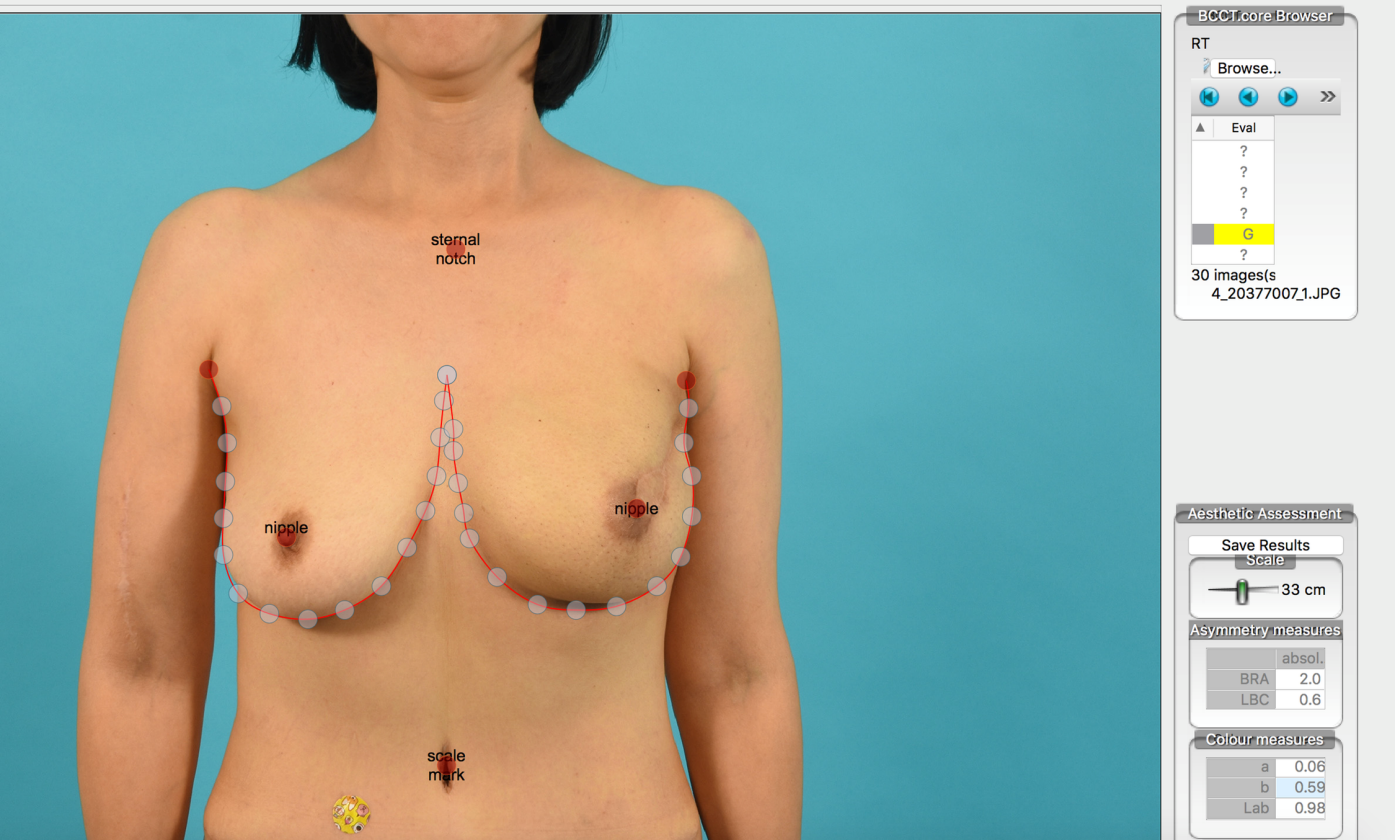
Breast cosmesis analysis. Breast cosmesis assessment using the Breast Cancer Conservative Treatment (BCCT)-core program.

Using the Breast Cancer Conservative Treatment (BCCT)-core program, cosmesis was analyzed by comparing photographs of the patients after radiation. Reference frontal photographic images obtained before the initiation of radiotherapy following breast reconstruction and photographs obtained one year after completion of the radiation treatment were analyzed (Fig 3). The cosmesis of the group not receiving radiation was also analyzed using the BCCT-core program (Fig 4).

### Statistical analysis

The chi-square test was performed on group-specific variables for propensity matching between the radiation group and the control group. The independent t-test method was used to compare the percentage differences in volume reduction between the two groups. For comparison of flap volumes before and after radiotherapy, we used the Wilcoxon signed rank test. Statistical significance was set at *P* < 0.05, if not otherwise specified.

## Results

Twenty patients underwent immediate breast reconstruction through TRAM flap and one patient underwent breast reconstruction through the DIEP flap in the study group. Patient age at the time of surgery was 46.7 years in the radiation group and 47.1 years in the non-radiation group. Body weight was 68.1 kg in the radiation group and 70.5 kg in the control group. Body mass index (BMI) was 26.3 kg/m^2^ in the radiation group and 26.9 kg/m^2^ in the control group. There was no statistically significant difference between the two groups with regard to the above characteristics (Table 1). Additionally, body weight and BMI were measured at each outpatient visit, and there was no statistically significant change between the 3-month postoperative and 18-month postoperative values. The mean total radiation dose in the radiation group was 52.4 Gy. Total flap loss was not observed in either group. Radiation-induced skin contracture and hyperpigmentation were observed in the study group (Table 2).

**Table 1:** Patient demographic characteristics.

		Radiation	Non-radiation	p-value
Age at surgery (years)		46.7	47.1	0.37
Weight (kg)		68.1	70.5	0.51
BMI (kg/m2)		26.3	26.9	0.76
Type of mastectomy	RM	2	0	
	MRM	5	2	
	TM	6	7	
	SSM	4	7	
	NSM	4	5	
Excised weight (g)		421	412	0.59
Insetted flap weight(g)		434	420	0.53
Total radiation dose (Gy)		52.4	0	

BMI, Body mass index; RM, radical mastectomy; MRM, modified radical mastectomy TM, total mastectomy; SSM, skin sparing mastectomy; NSM, nipple sparing mastectomy.

**Table 2:** Type and frequency of complications.

	Radiation		Non-radiation	
	n	%	n	%
Flap loss	0	0	0	0
Fat necrosis	7	33	3	14
Postoperative infection	4	19	2	10
Skin contracture	10	48	0	0
Hyperpigmentation	18	86	0	0
Asymmetry	9	43	2	10

The mean flap volume reduction over time was 12.3% when comparing the before and after irradiation volumes in the radiotherapy group. This was significantly higher (*P* < 0.01) than the 2.6% volume reduction observed in the control group. In addition, there was a statistically significant difference in the radiation group (Z = -4.015, *P* < 0.01) when comparing the volumes before and after radiation. In the control group, there were no significant differences in flap volumes between the 6-month and 12-month postoperative periods (Z = -1.356, *P* = 0.175) (Table 3).

**Table 3:** Assessment of flap volume changes.

Radiation		Average v value	Z & p value
	pre	396	Z = -4.015
	post1	355	
	pre-post (%)	12.3%*	p<0.01
Non-radiation			
	< 6m	401	Z = -1.356
	> 12m	393	
	pre-post (%)	2.60%	p = 0.175

* p<0.01.

In the BCCT-core program cosmetic evaluation, the radiation group showed excellent (11/21, 52.3%), good (7/21, 33.3%), and poor (3/21, 14.2%) results before radiation therapy and excellent (7/21, 33.3%), good (7/21, 33.3%), fair (5/21, 23.8%), and poor (2/21, 9.5%) results after radiation. In the control group, the short-term results (3–6 months) were excellent (10/21, 47.6%), good (8/21, 33.3%), fair (3/21, 14.2%), and poor (0/21, 0%), and the long-term results (12–18 months) were excellent (10/21, 47.6%), good (9/21, 33.3%), fair (2/21, 9.5%), and poor (0/21, 0%) (Table 4).

**Table 4:** Assessment of breast cosmesis using the Breast Cancer Conservative Treatment (BCCT)-core program.

Radiation		Pre (n, %)	Post (n/%)
	Excellent	11 (52.3)	7 (33.3)
	Good	7 (33.3)	7 (33.3)
	Fair	3 (14.2)	5 (23.8)
	Poor	0 (0)	2 (9.5%)
Non-radiation		< 6m (n, %)	> 12m (n/%)
	Excellent	10 (47.6)	10 (47.6)
	Good	8 (38)	9 (42.8)
	Fair	3 (14.2)	2 (9.5)
	Poor	0 (0)	0 (0)

## Discussion

Because of a decrease in resection size and an increase in the use of breast conserving therapies, an increasing number of patients with breast cancer are undergoing adjuvant radiation therapy. However, while radiation plays an important role in tumor regression, it also results in a moderate amount of damage to the surrounding normal tissues [13]. Complications from radiation including tissue contracture and necrosis are often the main symptoms experienced by patients with breast cancer who have undergone radiation therapy [8].

This is no different, even in the context of immediate breast reconstruction. Frequently, adjuvant radiotherapy is indicated postoperatively because of inadequate or positive surgical margins [4]. Additionally, if tumor spread to the axillary lymph nodes is suspected prior to surgery and the use of adjuvant radiation is anticipated, expander-based reconstruction is relatively contraindicated and most reconstructions are accomplished using an autologous flap. However, it remains challenging for the surgeon to predict the morphological and volumetric changes in the flap in response to radiation [14].

Previously, many studies have analyzed the complications of adjuvant radiation at the surgical site. In 2000 Tran et al. [15] reported the types of complications that occur after adjuvant radiation in patients with TRAM flaps; they found that hyperpigmentation, fat necrosis, skin and flap contracture, and loss of symmetry were the most frequent complications. Also in 2000, Hanks et al. [16] analyzed the acute effects of radiation therapy in patients undergoing a TRAM flap procedure and found that erythema was the most common symptom. Desquamation was also reported as a common complication, but no data related to long-term complications was presented. Several authors have reported the effects of postoperative radiation on flaps in various clinical reports, but no there have been no prior studies that quantitatively compared flap volume changes or aesthetic outcomes in these patients with those of patients not receiving radiation therapy.

The use of 3D CT to measure flap volume and reconstruct flap shape has been shown to be accurate in several previous studies [15–17]. In order to identify the exact boundaries of the flap on CT images, a board-certified radiologist and a plastic surgeon directly marked the boundaries of the flap. The flap volume was partitioned in 2 mm thick slices and modeled as a 3D structure using the Cavalieri principle. In addition, objective validation of the BCCT-core program, which was used in the present study, has been demonstrated in numerous studies. Cardoso et al. [18] reported that the BCCT-core program was an objective evaluation tool for the evaluation of breast cosmesis. Yu et al. [19] also reported the validity of the BCCT-core program in assessing the outcomes in patients undergoing breast conserving surgery and adjuvant radiation.

The results of this study will help surgeons determine the degree of overcorrection necessary when designing immediate breast reconstruction flaps in patients in whom adjuvant radiation therapy is anticipated. Since there was a 12.3% flap volume reduction after the completion of radiation, 14% overcorrection may be recommended for patients receiving adjuvant radiation therapy. However, a confirmatory study is also needed in the future. In addition, our findings can assist the surgeon in preoperatively precisely predicting those aesthetic changes that may occur after radiation therapy. When the surgeon has clinical information regarding the long-term changes in the surgical site after radiation therapy, immediate breast reconstruction can be performed in a manner that provides the best long-term results when adjuvant radiation therapy is anticipated.

In addition, the present study shows that there was no significant difference in the patients’ aesthetic results when radiation therapy was performed. Satisfactory cosmetic results can be obtained when reconstruction is performed with sufficient autologous tissue in patients undergoing adjuvant radiation therapy. Our aesthetic results compare favorably with those of previous clinical studies [20,21] of patients undergoing radiation therapy after expander-based reconstructions and provide a rationale for choosing autologous reconstruction options over expander-based reconstruction when adjuvant radiation is anticipated.

The present study has several limitations. First, the results did not compare the various locations in which volume reduction occurred after adjuvant radiation of the breast. However, previous studies have reported that tissue fibrosis and necrosis occur in the area of tissue irradiation, suggesting that volume loss occurs in the area of radiation administration. Second, most of the patients in this study underwent TRAM flap, and we did not find differences between the two surgeries in this study. However, it is difficult to apply these results directly to DIEP flap. Further research is needed on the volume changes of DIEP flaps after radiotherapy and whether there were differences in volume loss between these two surgeries. Third, as it was a retrospective study, we could not select one particular CT machine in advance. The diagnostic protocols of the patients were carried out per routine clinical care. However, the scanning protocols (e.g., CT scan parameters, radiation dose, dose modulation, resolution, filter, enhancement) were managed in almost the same manner in all devices by the radiologists. In particular, this was easier because both machines were products of the same company. Moreover, all the images used in the measurement were reconstructed by 5 mm images in the same manner as the post-processing using the multi-planar reformation function. In addition, the present study did not address the aesthetic results of expander-based breast reconstruction along with adjuvant radiation; additional studies are necessary to further evaluate the use of expander-based breast reconstruction and adjuvant radiation.

## Conclusions

When performing immediate autologous breast reconstruction, 14% volume overcorrection is recommended for patients in whom adjuvant radiation therapy is anticipated to improve aesthetic outcomes.

## Supporting information

S1 Table All de-identified data from this study. (XLSX)
